# Mechanical Synchrony and Myocardial Work in Heart Failure Patients With Left Bundle Branch Area Pacing and Comparison With Biventricular Pacing

**DOI:** 10.3389/fcvm.2021.727611

**Published:** 2021-08-20

**Authors:** Wen Liu, Chunqiang Hu, Yanan Wang, Yufei Cheng, Yingjie Zhao, Yang Liu, Shaoxin Zheng, Haiyan Chen, Xianhong Shu

**Affiliations:** ^1^Shanghai Institute of Cardiovascular Disease, Fudan University, Shanghai, China; ^2^Department of Echocardiography, Zhongshan Hospital, Fudan University, Shanghai, China; ^3^Shanghai Institute of Medical Imaging, Fudan University, Shanghai, China; ^4^Department of Cardiology, Sun Yat-sen Memorial Hospital, Sun Yat-sen University, Guangzhou, China; ^5^Department of Cardiology, Zhongshan Hospital, Fudan University, Shanghai, China

**Keywords:** cardiac resynchronization therapy, heart failure, left bundle branch block, myocardial work, mechanical synchronization

## Abstract

**Background:** Little is known about the efficacy of permanent left bundle branch area pacing (LBBAP) in delivering cardiac resynchronization therapy (CRT). This study aimed to evaluate the effect of LBBAP on mechanical synchronization and myocardial work (MW) in heart failure (HF) patients and to compare LBBAP with biventricular pacing (BVP).

**Methods:** This is a multicenter, prospective cohort study. From February 2018 to January 2021, 62 consecutive HF patients with reduced ejection fraction (LVEF ≤ 35%) and complete left bundle branch block (CLBBB) who underwent LBBAP or BVP were enrolled in this study. Echocardiograms and electrocardiograms and were conducted before and 3–6 months after implantation. Intra- and interventricular synchronization were assessed using two-dimensional speckle tracking imaging (2D-STI). The left ventricular pressure-strain loop was obtained by combining left ventricular strain with non-invasive blood pressure to evaluate mechanical efficiency.

**Results:** The echocardiographic response rates were 68.6 and 88.9% in the BVP and LBBAP groups, respectively. Left bundle branch area pacing resulted in significant QRS narrowing (from 177.1 ± 16.7 to 113.0 ± 18.4 ms, *P* < 0.001) and improvement in LVEF (from 29.9 ± 4.8 to 47.1 ± 8.3%, *P* < 0.001). The global wasted work (GWW) (410.3 ± 166.6 vs. 283.0 ± 129.6 mmHg%, *P* = 0.001) and global work efficiency (GWE) (64.6 ± 7.8 vs. 80.5 ± 5.7%, *P* < 0.001) were significantly improved along with shorter peak strain dispersion (PSD) (143.4 ± 45.2 vs. 92.6 ± 35.1 ms, *P* < 0.001) and interventricular mechanical delay (IVMD) (56.4 ± 28.5 vs. 28.9 ± 19.0 ms, *P* < 0.001), indicating its efficiency in improving mechanical synchronization. In comparison with BVP, LBBAP delivered greater improvement of QRS narrowing (−64.1 ± 18.9 vs. −32.5 ± 22.3 ms, *P* < 0.001) and better mechanical synchronization and efficiency.

**Conclusions:** Left bundle branch area pacing was effective in improving cardiac function, mechanical synchronization, and mechanical efficiency and may be a promising alternative cardiac resynchronization therapy.

## Introduction

Biventricular pacing (BVP) is a traditional method of cardiac resynchronization therapy (CRT), and long-term studies have shown a significant reduction in mortality in heart failure (HF) patients with complete left bundle branch block (CLBBB) (1, 2). However, BVP causes non-physiological ventricular activation patterns with a prolonged paced QRS duration (QRSd) and up to 30% of patients appear to achieve no clinical benefit (3, 4).

As first published in 2017 (5), Huang et al. reported that permanent left bundle branch area pacing (LBBAP) can effectively normalize the left bundle branch block (LBBB) with a stable pacing threshold and improve cardiac function in HF patients during follow-up (6), serving as a promising alternative to BVP. To date, several clinical studies have demonstrated the feasibility of LBBAP in HF patients (7, 8). However, the existing data are not sufficient, and little is known about the effect on mechanical synchronization and myocardial work (MW).

Previous studies have proven the role of two-dimensional speckle tracking imaging (2D-STI) in evaluating intra- and interventricular dyssynchrony (9, 10). The pressure-strain loop, which has emerged as a novel non-invasive method developed from STI, is more effective in quantitatively assessing mechanical synchrony and mechanical efficiency associated with left ventricular pressure (11, 12). Therefore, we designed this study to evaluate the efficacy of LBBAP in advanced HF patients with CLBBB using 2D-STI combined with MW. A preliminary comparison between LBBAP and BVP was also performed in our study.

## Materials and Methods

### Study Design

This study is a multicenter, prospective cohort study. Sixty-two consecutive patients referred for CRT according to the 2016 European Society of Cardiology (ESC) guidelines (13) were recruited from February 2018 to January 2021 at Zhongshan Hospital of Fudan University, Sun Yat-Sen Memorial Hospital and the First Affiliated Hospital of Wenzhou Medical University. Informed written consent was obtained from all participants under the approval of the ethics committees of participating hospitals.

The inclusion criteria were as follows: (a) symptomatic patients with LVEF ≤ 35% despite optimal medical treatment for at least 3 months; (b) CLBBB morphology and QRSd ≥130 ms; and (c) age ≥18 years old. The exclusion criteria were as follows: (a) narrow QRS or non-LBBB morphology; (b) absence of clinical follow-up or poor condition of the acoustic window; and (c) life expectancy < 1 year. An echocardiographic response was defined as a ≥10% absolute increase in LVEF compared with the baseline, and a super-response was defined as an absolute increase ≥20% in LVEF compared with the baseline or LVEF ≥50% at follow-up.

### Implantation Procedure and Programming

#### Left Bundle Branch Area Pacing

Left bundle branch area pacing was performed with an LBBAP pacing lead (Model 3830; SelectSecure, Medtronic, Inc., Minneapolis, MN) supported by a delivery catheter (C315His; Medtronic, Inc.) under a 30° right anterior oblique (RAO) fluoroscopic view. The left bundle branch was located between the tricuspid valve, non-coronal sinus, and right coronary sinus. When the pacing lead was first applied on the right side of the interventricular septum, the paced QRS morphology demonstrated a “w” shape with a notch at the nadir of the QRS in lead V1. The sheath with the LBBAP pacing lead was then screwed counterclockwise into the interventricular septum, usually 10–20 mm away from the His bundle region. Twelve-lead ECG and intracardiac electrograms were simultaneously recorded and applied to identify the ideal pacing site. During the advancement process, the notch at the nadir of the QRS gradually moves up to the end of the QRS wave. Once the R wave appears at the terminal of QRS in surface lead V1, indicating the right bundle branch block (RBBB) pacing morphology, the lead advancement process should cease. The position was reconfirmed by intra-sheath radiography or trans-thoracic echocardiography. The ideal pacing site should meet the following criteria: (1) the QRSd narrows significantly, and the LBBB can be partly or completely normalized; and (2) the fast peak left ventricular activation time (LVAT) measured in leads V4–V6 is constant regardless of high or low output. The coronary sinus-left ventricular (CS-LV) lead was implanted as a backup for resynchronization therapy. If LBBAP could effectively normalize LBBB or narrow QRS ≤ 140 ms, devices were set in LBBAP only. Otherwise, sequential pacing of the LBBAP and CS-LV lead was programmed, and optimal narrow QRS was obtained by adjusting the LV–RV (V–V) interval. The right atrial leads (Boston 4480) were implanted into the right atrial appendage. The AV interval was optimized according to surface ECG in full consideration of the conduction delay between the left bundle branch pacing pulse and the QRS wave (20–30 ms).

#### Biventricular Pacing

The left ventricular lead (Boston 4675) was inserted into the lateral, posterolateral, or posterior veins, preferably after retrograde coronary venography. The right ventricular (Boston 0693) and atrial leads (Boston 7736) were fixed on the right ventricular septum or apex and right atrium, respectively. The A–V and V–V intervals were routinely adjusted during the follow-up to achieve optimized narrowing of the QRSd.

### Clinical Evaluation and Follow-Up

#### Clinical Data

Medical history, physical examinations, New York Heart Association (NYHA) functional classification, 12-lead electrocardiogram, and echocardiograph were evaluated at baseline and at 3–6 months during follow-up. QRS duration was measured by a standard 12-lead electrocardiogram (ECG) from the starting point of the Q wave to the end point of the S wave, while paced QRSd was measured from the pacing stimulus to the end point of the S wave.

#### Echocardiographic Parameters

Echocardiography was performed using GE Vivid E9 or E95 ultrasound equipment (GE Company, USA) by experienced senior echocardiography physicians. Standard echocardiogram indices, including left atrial diameter (LAD), left ventricular end systolic/diastolic diameter (LVESD/LVEDD), and left ventricular end-systolic/diastolic volume (LVEDV/LVESV), were acquired. Left ventricular ejection fraction (LVEF) was evaluated by two-dimensional biplane Simpson's method. Echocardiographic images of the apical two-, three-, and four-chamber views were collected continuously for at least five cardiac cycles, and the mean frame rate of images was 60 ± 5 frames/s. Non-invasive blood pressure recordings representing left ventricular pressure were taken by a brachial artery sphygmomanometer at the same time.

The qualification of MW was conducted by software (Echopac V.202, GE) using the AFI package and analyzed according to the following steps. First, the duration of isovolumic and ejection phases was defined by valvular timing (the opening and closing time of mitral and aortic valve) according to pulse wave Doppler imaging. Then, global myocardial longitudinal peak strain (GLS) was calculated by speckle tracking analysis using standard apical views (long-axis, two-chamber, and four-chamber) (14). Finally, the LV pressure-strain loop was constructed automatically with a combination of LV strain and non-invasive blood pressure measurements adjusted by the duration of the isovolumic and ejection phases (15). Global constructive work (GCW, work performed by systolic shortening and myocardial lengthening in the isovolumetric relaxation phase); global wasted work (GWW, work performed by systolic lengthening and myocardial shortening in the isovolumetric relaxation phase); global work efficiency (GWE, the ratio between constructive work and the sum of wasted and constructive work); and the global work index (GWI, work performed during the period from mitral valve closure to mitral valve opening) were acquired. Myocardial work (MW), myocardial work efficiency (MWE), wasted work (WW) were calculated for each LV segment. Segmental work was calculated as the average of basal and mid segments in the apical four-chamber view. The lateral–septal MW difference was acquired to evaluate distribution of regional MW.

Segment systolic time to peak longitudinal systolic strain was assessed for every participant. The difference in systolic times to peak 2-D strain between segments was calculated to reflect intraventricular mechanical synchronization. Peak strain dispersion (PSD) is defined as the standard deviation of time to peak longitudinal systolic strain of LV segments. Interventricular mechanical delay (IVMD) was measured to reflect the mechanical synchronization between the left and right ventricles evaluated by pulse wave Doppler imaging.

### Statistics Analysis

Statistical analysis was performed with SPSS 13.0 software (SPSS Inc., Chicago, Illinois). Continuous variables are presented as the mean ± standard deviation. Categorical variables are expressed as percentages. Differences between groups were assessed with chi-square analysis for categorical variables and *t*-tests or non-parametric tests for continuous data at baseline. Paired samples *t*-tests or non-parametric tests were used to compare the echocardiographic parameters at baseline and follow-up. A linear mixed-effects model was used to investigate the independent association between different pacing strategies and changes in echocardiographic outcomes. A *P*-value < 0.05 was indicative of statistical significance.

## Results

### Study Population

A total of 62 advanced HF patients (mean age, 64.8 ± 8.5 years; 54.8% male) with CLBBB were enrolled between February 2018 and January 2021 at the three centers. The clinical characteristics of all patients are summarized in Table 1. Among them, 35 patients (mean age, 64.3 ± 8.4 years; 57.1% male) underwent BVP, 27 patients (mean age, 65.5 ± 8.8 years; 51.9% male) received LBBAP. Medical treatment was optimized for at least 3 months before implantation. There was no significant difference between the LBBAP and BVP groups in baseline demographics, medical history, comorbidities, electrocardiography, or echocardiography parameters and myocardial work indices (Tables 1, **4**).

**Table 1:** Baseline characteristics of the patients.

**Variables**	**BVP (*n* = 35)**	**LBBAP (*n* = 27)**	***P*-value**
Male gender, *n* (%)	20 (57.1%)	14 (51.9%)	0.678
Age (years)	64.3 ± 8.4	65.5 ± 8.8	0.606
follow-up (months)	4.4 ± 1.4	4.0 ± 1.4	0.220
Heart rate (beats/min)	73.7 ± 14.6	72.9 ± 12.0	0.805
Intrinsic QRSd (ms)	168.8 ± 16.8	177.1 ± 16.7	0.057
**NYHA functional class**	2.8 ± 0.6	3.0 ± 0.5	0.326
NYHA II, *n* (%)	9 (25.7%)	4 (14.8%)	
NYHA III, *n* (%)	23 (65.7%)	20 (74.1%)	
NYHA IV, *n* (%)	3 (8.6%)	3 (11.1%)	
NT-proBNP (pg/ml)	2602.0 ± 3245.1	2220.5 ± 3712.5	0.848
LVEF (%)	29.5 ± 4.9	29.9 ± 4.8	0.689
SBP (mmHg)	120.3 ± 14.7	121.7 ± 15.6	0.680
DBP (mmHg)	72.2 ± 7.4	71.2 ± 10.5	0.666
**Comorbidity**			
Renal insufficiency, *n* (%)	1 (2.9%)	2 (7.4%)	0.817
Diabetes, *n* (%)	8 (22.9%)	9 (33.3%)	0.359
Hypertension, *n* (%)	16 (45.7%)	11 (40.7%)	0.695
Ischemic Etiology *n* (%)	8 (22.9%)	7 (25.9%)	0.735
Paroxysmal Af or AF *n* (%)	4 (11.4%)	3 (11.1%)	1.000
**Medication**			
aldactone, *n* (%)	33 (94.3%)	20 (74.1%)	0.061
ACEI/ARB/ARNI, *n* (%)	33 (94.3%)	24 (88.9%)	0.762
Beta-blockers, *n* (%)	32 (91.4%)	24 (88.9%)	1.000
amiodarone, *n* (%)	4 (11.4%)	2 (7.4%)	0.922

*NYHA, New York heart association; LVEF, left ventricular ejection fraction; SBP, systolic blood pressure; DBP, diastolic blood pressure; Af, atrial fibrillation; AF, atrial flutter; ACEI, angiotensin-converting enzyme inhibitor; ARB, angiotensin II receptor blocker; ARNI, angiotensin receptor neprilysin inhibitor*.

*p < 0.05.

### Procedure Outcomes of LBBAP

Left bundle branch area pacing implantation was successful in 27 of the 34 patients (79.4%), with full correction of LBBB or narrow QRS ≤ 140 ms. Sequential pacing of the LBBAP and CS-LV was programmed to achieve further narrowing QRSd in 5 of 34 patients (14.7%). Left bundle branch area pacing implantation failed in 5.9% (2/34) because of an inability to achieve conduction system capture. During the implantation process, 4 of 32 (12.5%) patients had transient III° atrioventricular conduction blocks, but all recovered after their operations. No ventricular septal ruptures were observed during the procedures.

### Cardiac Function of LBBAP

**Figure 1:**
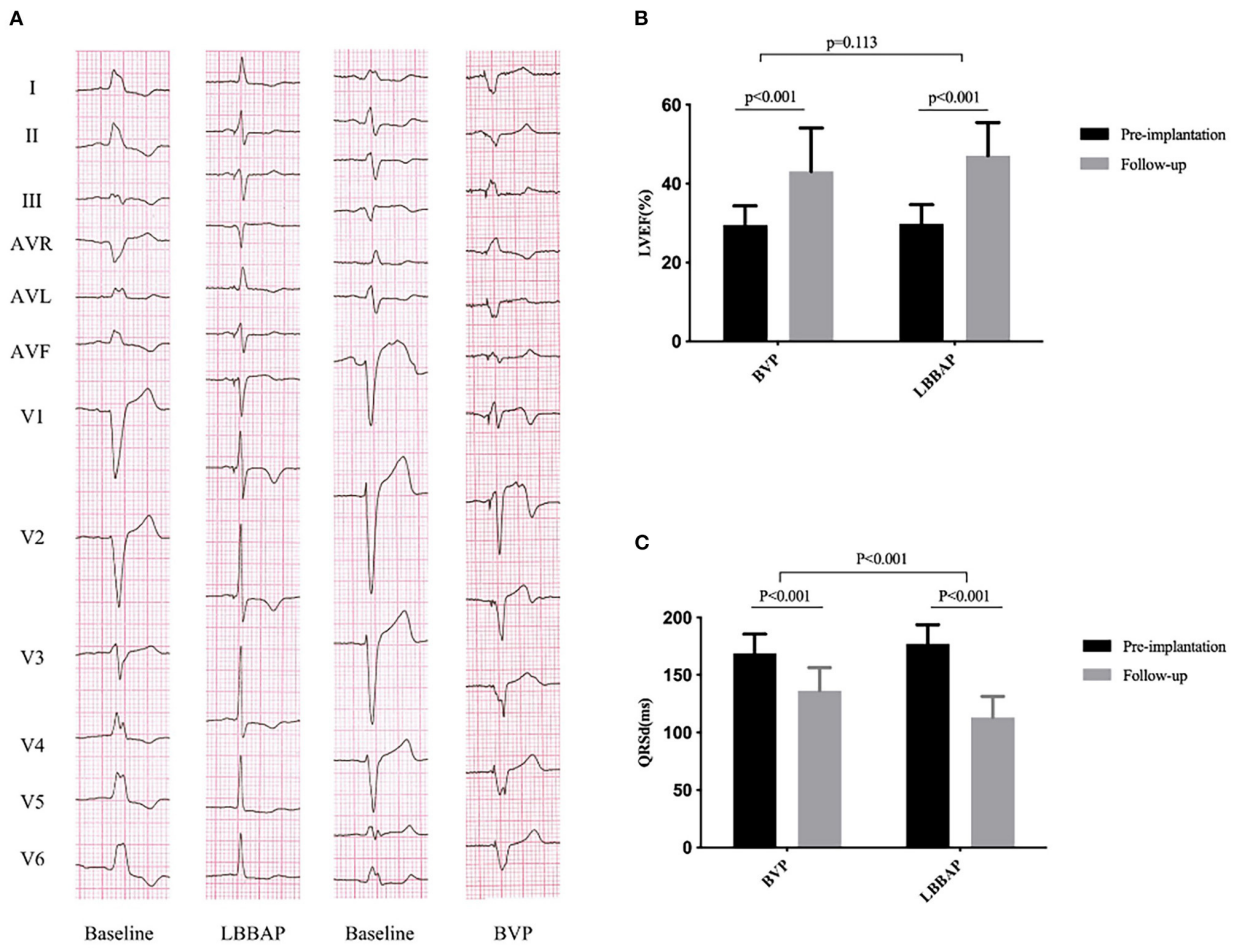
QRS duration and cardiac function at baseline and follow-up in the BVP and LBBAP groups. **(A)** Twelve-lead ECG from baseline to follow-up; **(B)** left ventricular ejection fraction (LVEF) from baseline to follow-up; **(C)** QRS duration (QRSd) from baseline to follow-up.

During a short-term follow-up (mean, 4.0 ± 1.4 months; range from 3 to 6 months), the NYHA functional class was improved from 3.0 ± 0.5 at baseline to 1.6 ± 0.6 (*P* < 0.001), with significantly improved cardiac function (LVEF: from 29.9 ± 4.8 to 47.1 ± 8.3%, *P* < 0.001; GLS: from −5.6 ± 1.9 to −9.9 ± 2.3%, *P* < 0.001). An echocardiographic response, defined as ≥10% absolute improvement in LVEF compared with the baseline was observed in 24 of 27 patients (88.9%). Super-response (absolute increase ≥20% of LVEF or LVEF ≥50%) was identified in 12 of 27 patients (44.4%). The echocardiographic response rate in non-ischemic cardiomyopathy patients and ischemic patients was 90.0% (18/ 20), 85.7% (6/7), respectively. There was a significant reduction in left ventricular end-systolic diameter and volume (LVESD: from 56.6 ± 7.8 to 45.0 ± 7.5 mm, *P* < 0.001; LVESV: from 141.4 ± 40.6 to 72.6 ± 31.5 ml, *P* < 0.001) (Table 2; Figure 1).

**Table 2:** Echocardiographic data in the LBBAP group at baseline and follow-up.

**Variables**	**Baseline**	**Follow-up**	***P*-value**
NYHA functional class	3.0 ± 0.5	1.6 ± 0.6	< 0.001^*^
GLS (%)	−5.6 ± 1.9	−9.9 ± 2.3	< 0.001^*^
LVEF (%)	29.9 ± 4.8	47.1 ± 8.3	< 0.001^*^
LVEDD (mm)	67.9 ± 6.6	57.7 ± 4.9	< 0.001^*^
LVESD (mm)	56.6 ± 7.8	45.0 ± 7.5	< 0.001^*^
LVEDV (ml)	200.8 ± 49.6	133.6 ± 45.8	< 0.001^*^
LVESV (ml)	141.4 ± 40.6	72.6 ± 31.5	< 0.001^*^
LAD (mm)	46.5 ± 5.1	41.7 ± 5.9	< 0.001^*^
PASP (mmHg)	41.6 ± 13.5	34.5 ± 6.3	0.010^*^
TAPSE (mm)	16.3 ± 2.4	17.9 ± 1.7	0.013^*^

*NYHA, New York heart association; GLS, global longitudinal strain; LVEF, left ventricular ejection fraction; LVEDD, left ventricular end-diastolic diameter; LVESD, left ventricular end-systolic diameter; LVEDV, left ventricular end-diastolic volume; LVESV, left ventricular end-systolic volume; PASP, pulmonary arterial systolic pressure; TAPSE, Tricuspid annulus systolic displacement; LAD, left atrial diameter*.

* *p < 0.05*.

### Mechanical Synchronization of LBBAP

**Figure 2:**
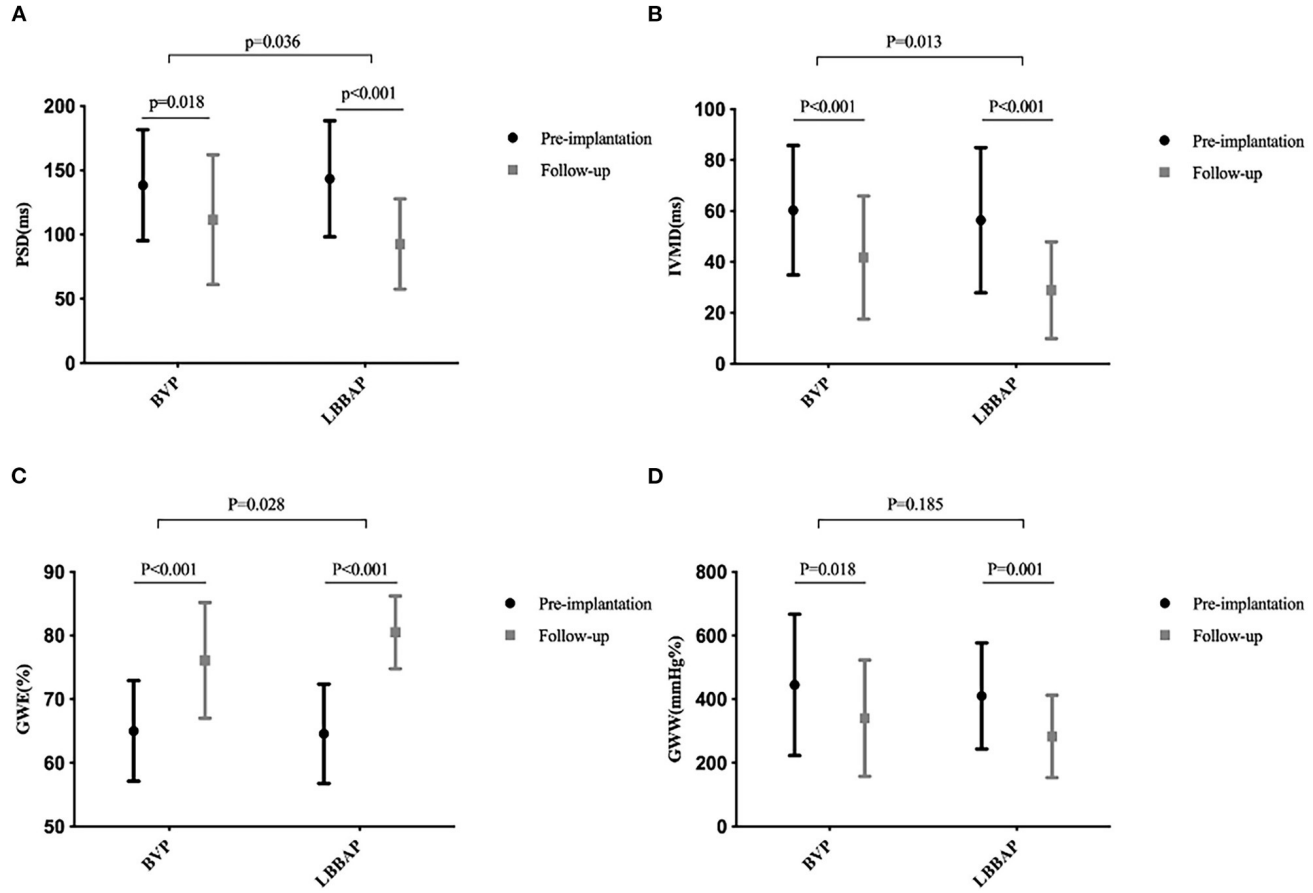
Mechanical synchronization at baseline and follow-up in the BVP and LBBAP groups. **(A)** Peak strain dispersion (PSD) from baseline to follow-up; **(B)** interventricular mechanical delay (IVMD) from baseline to follow-up; **(C)** global work efficiency (GWE) from baseline to follow-up; **(D)** global wasted work (GWW) from baseline to follow-up.

As shown in Table 3, the QRSd was significantly shortened after LBBAP implantation compared with that at baseline (177.1 ± 16.7 vs. 113.0 ± 18.4 ms, *P* < 0.001). Left bundle branch area pacing significantly shortened the duration of IVMD (from 56.4 ± 28.5 to 28.9 ± 19.0 ms, *p* < 0.001) and PSD (from 143.4 ± 45.2 to 92.6 ± 35.1 ms, *p* < 0.001) during the follow-up. Postoperatively, the 17-segment maximum time difference to peak 2-D strain (from 436.3 ± 166.2 to 284.1 ± 164.2 ms, *p* < 0.001) was significantly shortened in the LBBAP group. There was significant improvement in the time difference to peak 2-D strain between basal anteroseptal vs. posterior segments (143.0 ± 113.7 vs. 104.0 ± 94.7 ms, *P* = 0.038) and basal anterior vs. inferior segments (131.6 ± 129.5 vs. 117.7 ± 110.3 ms, *P* = 0.016) (Table 3; Figure 2).

**Table 3:** Asynchronization status in the LBBAP group at baseline and follow-up.

**Variables**	**Baseline**	**Follow -up**	***P*-value**
QRSd (ms)	177.1 ± 16.7	113.0 ± 18.4	< 0.001^*^
IVMD (ms)	56.4 ± 28.5	28.9 ± 19.0	< 0.001^*^
PSD (ms)	143.4 ± 45.2	92.6 ± 35.1	< 0.001^*^
Segment maximum time difference to peak 2-D strain (ms)	436.3 ± 166.2	284.1 ± 164.2	< 0.001^*^
Basal anteroseptal vs. posterior segments (ms)	143.0 ± 113.7	104.0 ± 94.7	0.038^*^
Basal anterior vs. inferior segments (ms)	131.6 ± 129.5	117.7 ± 110.3	0.016^*^
Basal septal vs. lateral segments (ms)	178.0 ± 119.0	129.0 ± 139.7	0.174

*IVMD, interventricular mechanical delay; PSD, peak strain dispersion*.

* *p < 0.05*.

### Myocardial Work of LBBAP

**Figure 3:**
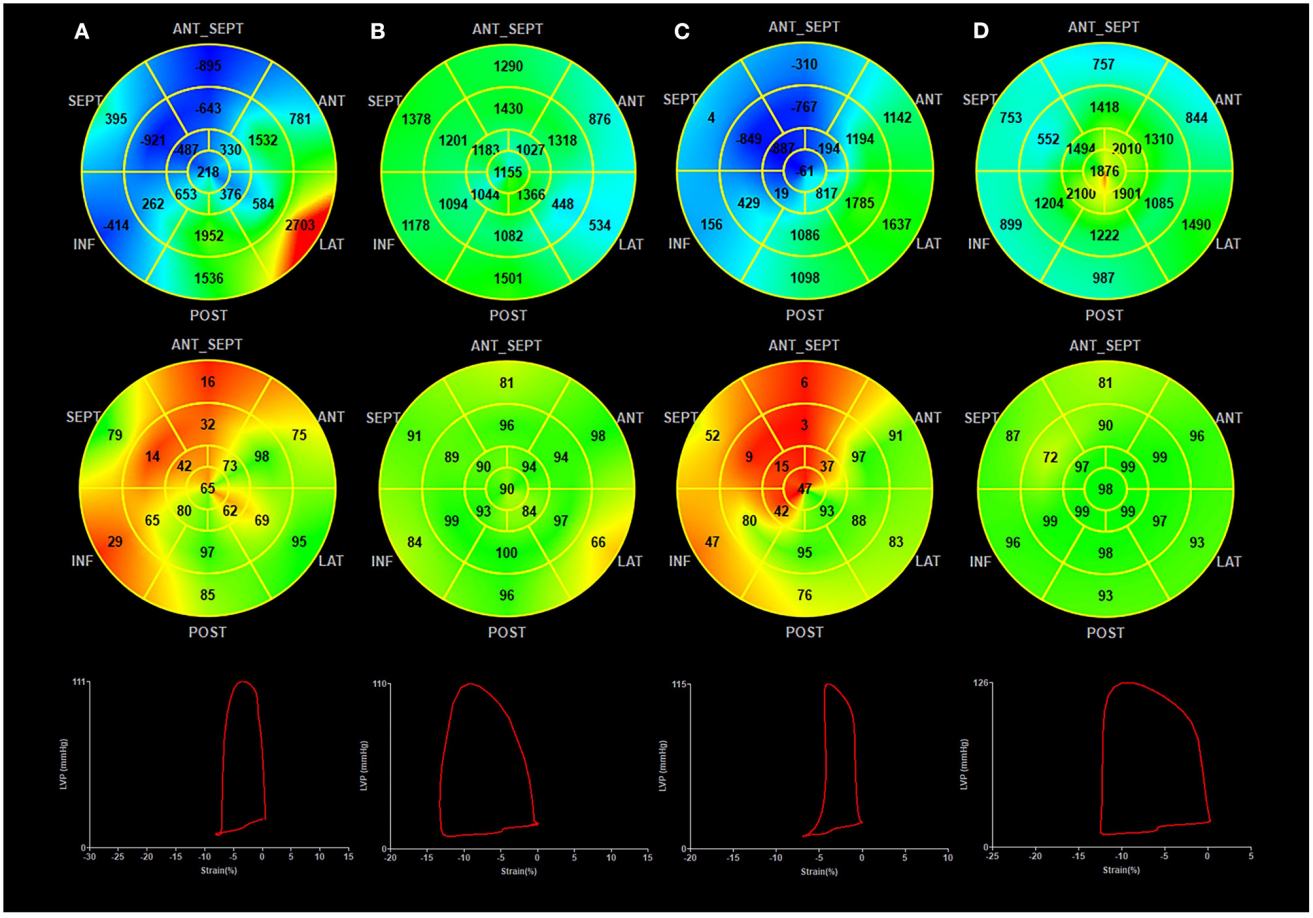
Myocardial work at baseline and follow-up in the BVP and LBBAP groups. **(A)** BVP at baseline; **(B)** BVP at follow-up; **(C)** LBBAP at baseline; **(D)** LBBAP at follow-up; (Top Panel) Seventeen-segment bull's-eye of myocardial work index (negative work in blue, normal in green, and areas of high myocardial work coded in red); (Middle Panel) Seventeen-segment bull's-eye of myocardial work efficiency (high efficiency in green, low efficiency in red); (Bottom panel) Pressure-strain loops.

**Figure 4:**
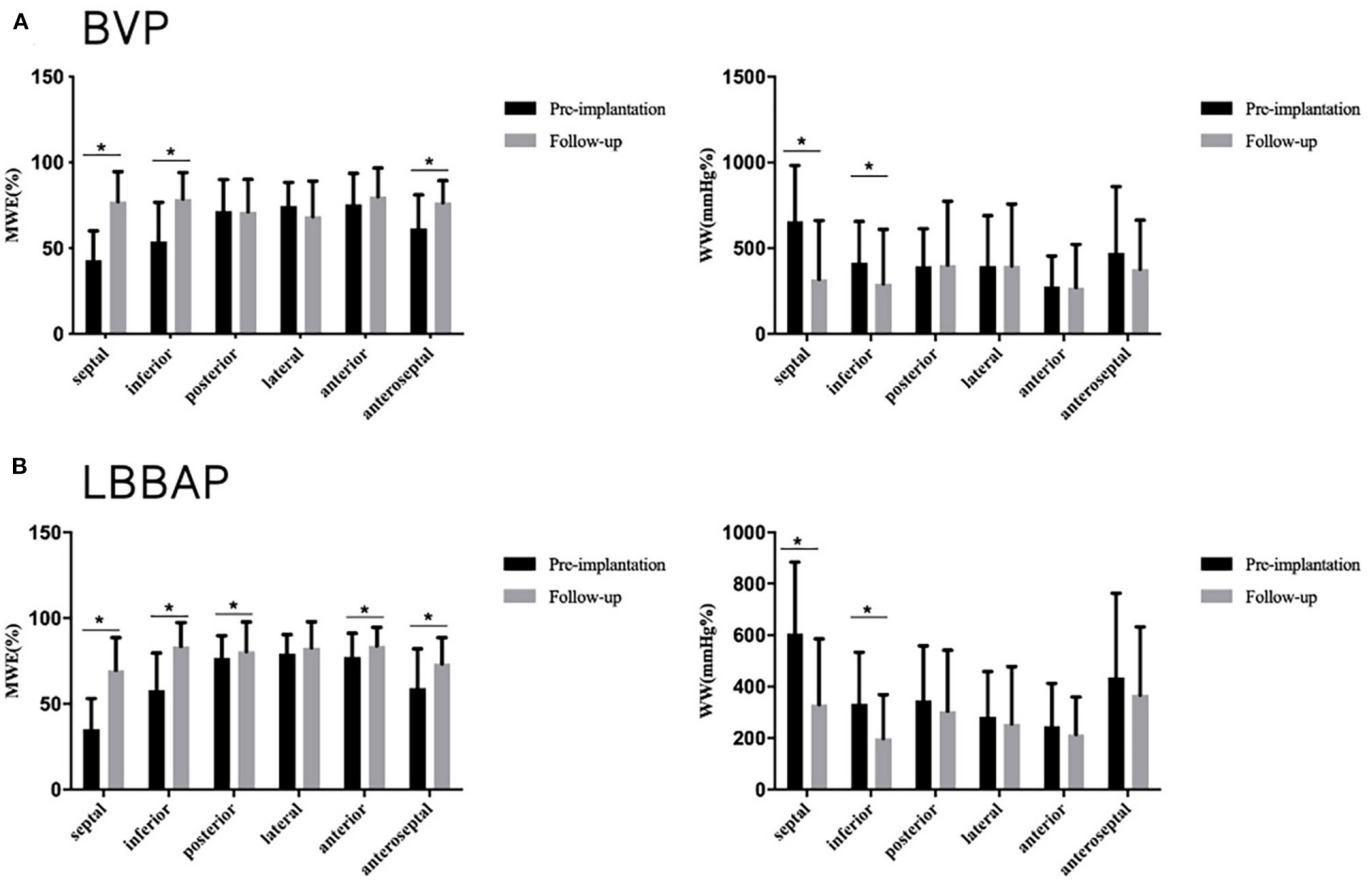
Segmental myocardial work at baseline and follow-up in the BVP and LBBAP groups. **(A)** Segmental myocardial work (MWE) and segmental wasted work (WW) at baseline and follow-up in the BVP group; **(B)** Segmental myocardial work (MWE) and segmental wasted work (WW) at baseline and follow-up in the LBBAP group. *p < 0.05.

During the follow-up, GWE was improved from 64.6 ± 7.8% at baseline to 80.5 ± 5.7% (*P* < 0.001), with a significant reduction in GWW (from 410.3 ± 166.6 to 283.0 ± 129.6 mmHg%, *P* < 0.001). Global constructive work and GWI were also significantly ameliorated (from 836.0 ± 198.4 to 1321.6 ± 371.4 mmHg%, *P* < 0.001; from 485.0 ± 200.7 to 1093.3 ± 343.2 mmHg%, *P* < 0.001) (Table 4; Figure 3). As with segmental myocardial work, segmental MWE was significantly improved in the septal (from 35.3 ± 17.8 to 69.6 ± 19.0%, *P* = 0.001), inferior (from 57.9 ± 21.7 to 83.6 ± 13.7 %, *P* = 0.001), posterior (from 76.7 ± 13.0 to 80.6 ± 17.1%, *P* = 0.045), anterior (from 77.3 ± 13.7 to 83.8 ± 10.7%, *P* = 0.027), and anteroseptal (from 59.2 ± 22.8 to 73.3 ± 15.3%, *P* = 0.016) segments. There was a trend toward a reduction in mean segmental WW in every segment and it was significantly reduced in the septal (from 607.1 ± 276.5 to 330.8 ± 254.3 mmHg%, *P* < 0.001), inferior (from 333.7 ± 199.8 to 198.6 ± 169.8 mmHg%, *P* = 0.009) segments compared with the baseline. The MW differences between the lateral and septal segments were significantly reduced at the time of follow-up (from 1172.2 ± 563.5 to 633.1 ± 596.6 mmHg%, *P* = 0.001) (Table 4; Figure 4).

**Table 4:** Global and segmental myocardial work in the LBBAP and BVP groups at baseline and follow-up.

	**BVP**		***P*-value**	**LBBAP**		***P*-value**	***P*-value**
	**Baseline**	**Follow-up**	**(BVP baseline**	**Baseline**	**Follow-up**	**(LBBAP baseline**	**(Baseline BVP**
			**vs. follow-up)**			**vs. follow-up)**	**vs. LBBAP)**
GWE (%)	65.0 ± 7.9	76.1 ± 9.1	< 0.001^*^	64.6 ± 7.8	80.5 ± 5.7	< 0.001^*^	0.826
GWI (mmHg%)	526.5 ± 311.4	877.2 ± 388.1	< 0.001^*^	485.0 ± 200.7	1093.3 ± 343.2	< 0.001^*^	0.938
GCW (mmHg%)	897.3 ± 386.6	1176.9 ± 421.3	< 0.001^*^	836.0 ± 198.4	1321.6 ± 371.4	< 0.001^*^	0.870
GWW (mmHg%)	445.1 ± 222.0	340.5 ± 182.7	0.018 ^*^	410.3 ± 166.6	283.0 ± 129.6	0.001^*^	0.938
**MWE (%)**							
Septal segment	42.9 ± 17.1	77.2 ± 17.4	0.001^*^	35.3 ± 17.8	69.6 ± 19.0	0.001^*^	0.093
inferior segment	53.9 ± 22.8	78.7 ± 15.3	0.001^*^	57.9 ± 21.7	83.6 ± 13.7	0.001^*^	0.485
Posterior segment	71.6 ± 18.3	71.2 ± 18.9	0.889	76.7 ± 13.0	80.6 ± 17.1	0.045^*^	0.341
Lateral segment	74.7 ± 13.6	68.4 ± 20.7	0.109	79.1 ± 11.2	82.7 ± 15.1	0.254	0.173
Anterior segment	75.5 ± 18.0	80.1 ± 16.6	0.252	77.3 ± 13.7	83.8 ± 10.7	0.027^*^	0.932
Anteroseptal segment	61.6 ± 19.5	76.5 ± 12.8	0.001^*^	59.2 ± 22.8	73.3 ± 15.3	0.016^*^	0.662
**WW (mmHg%)**							
Septal segment	657.3 ± 324.6	317.6 ± 342.5	0.001^*^	607.1 ± 276.5	330.8 ± 254.3	< 0.001^*^	0.522
Inferior segment	414.0 ± 241.5	291.8 ± 317.4	0.005^*^	333.7 ± 199.8	198.6 ± 169.8	0.009^*^	0.110
Posterior segment	394.1 ± 219.3	402.4 ± 370.9	0.888	345.9 ± 212.6	304.3 ± 236.8	0.212	0.262
Lateral segment	395.0 ± 293.8	397.0 ± 359.8	0.974	283.1 ± 175.7	255.4 ± 222.4	0.479	0.110
Anterior segment	277.1 ± 177.4	270.0 ± 252.4	0.900	246.4 ± 166.7	214.4 ± 145.4	0.337	0.491
Anteroseptal segment	472.3 ± 385.5	378.0 ± 284.9	0.207	436.0 ± 327.3	368.2 ± 263.6	0.319	0.848
**Segmental MW (mmHg%)**							
Septal MW	−42.9 ± 377.5	886.8 ± 522.6	< 0.001^*^	−151.0 ± 294.0	695.9 ± 510.6	< 0.001^*^	0.225
Lateral MW	834.5 ± 555.5	521.6 ± 521.3	0.005^*^	1021.2 ± 466.5	1329.0 ± 535.2	0.007^*^	0.165
Lateral-septal difference	877.4 ± 687.4	−365.2 ± 644.9	< 0.001^*^	1172.2 ± 563.5	633.1 ± 596.6	0.001^*^	0.076

*GWE, global work efficiency; GWI, global work index; GCW, global constructive work; GWW, global wasted work; MWE, myocardial work efficiency; WW, wasted work; MW, myocardial work*.

* *p < 0.05*.

### Comparison Between LBBAP and BVP

A linear mixed-effects model was used to compare the parameters between LBBAP and BVP with consideration of baseline variables and follow-up duration. The echocardiographic response rate, defined as a ≥10% absolute increase in LVEF compared with the baseline, was 68.6 and 88.9% in the BVP and LBBAP groups, respectively. A relatively low no-response rate was observed in the LBBAP (11.1 vs. 31.4%) group compared with the BVP group. During the short-term follow-up, improvement of overall NYHA functional class in LBBAP was greater than that in BVP group (−1.6 ± 0.6 vs. −0.9 ± 0.8, *P* = 0.001). Although the degree of LVEF improvement did not achieve any significant difference, GLS was better ameliorated in the LBBAP (−4.3 ± 2.2 vs. −2.3 ± 2.6%, *P* < 0.001) group than in the BVP group (Table 5). Significantly narrowed QRSd were achieved in the LBBAP group (−64.1 ± 18.9 vs. −32.5 ± 22.3 ms, *p* < 0.001) than in the BVP group. The IVMD and PSD, which reflect interventricular and intraventricular mechanical synchrony, were better improved in the LBBAP group than in the BVP group (−27.4 ± 28.7 vs. −18.6 ± 27.9 ms, *p* = 0.013; −50.9 ± 56.8 vs. −26.9 ± 63.9 ms, *p* = 0.036).

**Table 5:** Comparison between BVP and LBBAP groups.

**Variables**	**Change**		**Difference**	***P*-value for linear**
			**(95% CI)**	**mixed-effects model ^#^**
	**BVP**	**LBBAP**		
**Cardiac function**				
^Δ^NYHA functional class	−0.9 ± 0.8	−1.6 ± 0.6	−0.7 (−1.1,−0.3)	0.001^*^
^Δ^GLS (%)	−2.3 ± 2.6	−4.3 ± 2.2	−2.0 (−3.2,−0.7)	< 0.001^*^
^Δ^LVEF (%)	13.7 ± 11.5	17.2 ± 9.3	3.6 (−1.9,9.0)	0.113^*^
**Synchronization**				
^Δ^QRSd (ms)	−32.5 ± 22.3	−64.1 ± 18.9	−31.6 (−42.3,−20.9)	< 0.001^*^
^Δ^IVMD (ms)	−18.6 ± 27.9	−27.4 ± 28.7	−8.9 (−23.3,5.6)	0.013^*^
^Δ^PSD (ms)	−26.9 ± 63.9	−50.9 ± 56.8	−23.9 (−55.2,7.3)	0.036^*^
**Global myocardial work**				
^Δ^GWE (%)	11.1 ± 10.6	15.9 ± 8.9	4.8 (−0.3,9.8)	0.028^*^
^Δ^GWW (mmHg%)	−104.6 ± 248.9	−127.3 ± 181.8	−22.7 (−136.6,91.2)	0.185
^Δ^GWI (mmHg%)	350.7 ± 352.4	608.3 ± 353.0	257.5 (76.8,438.2)	0.007^*^
^Δ^GCW (mmHg%)	279.6 ± 388.8	485.5 ± 359.5	205.9 (13.1,398.8)	0.031^*^
**Segmental MWE (%)**				
^Δ^Septal segment	34.3 ± 26.7	34.4 ± 23.9	0.0 (−13.1,13.1)	0.121
^Δ^Inferior segment	24.8 ± 23.1	25.7 ± 26.3	0.9 (−11.7,13.5)	0.238
^Δ^Posterior segment	−0.4 ± 17.5	3.8 ± 18.0	4.2 (−4.8,13.3)	0.068
^Δ^Lateral segment	−6.4 ± 22.9	3.5 ± 15.7	9.9 (0.1,19.7)	0.006^*^
^Δ^Anterior segment	4.6 ± 23.1	6.5 ± 14.5	2.0 (−7.6,11.6)	0.351
^Δ^Anteroseptal segment	14.9 ± 20.8	14.1 ± 25.8	−0.8 (−12.7,11.0)	0.433

*Abbreviations see Tables 2–4*.

# *Adjusted by baseline QRSd, baseline LVEF, baseline self-parameter, and follow-up duration*.

* *p < 0.05*.

Compared with those in the BVP group, patients who received LBBAP had greater improvements in GWE (15.9 ± 8.9 vs. 11.1 ± 10.6%, *P* = 0.028), GWI (608.3 ± 353.0 vs. 350.7 ± 352.4 mmHg%, *P* = 0.007), and GCW (485.5 ± 359.5 vs. 279.6 ± 388.8 mmHg%, *P* = 0.031). As with segmental myocardial work, segmental MWE was significantly improved in all LV segments except for the lateral segment in the LBBAP group. Biventricular pacing was poor at improving MWE in the posterior (from 71.6 ± 18.3 to 71.2 ± 18.9%, *P* = 0.889), lateral (from 74.7 ± 13.6 to 68.4 ± 20.7%, *P* = 0.109), and anterior segments (from 75.5 ± 18.0 to 80.1 ± 16.6%, *P* = 0.252). Wasted work was significantly ameliorated in the septal (LBBAP: from 607.1 ± 276.5 to 330.8 ± 254.3 mmHg%, *P* < 0.001; BVP: from 657.3 ± 324.6to 317.6 ± 342.5 mmHg%, *P* = 0.001), and inferior (LBBAP: from 333.7 ± 199.8 to 198.6 ± 169.8 mmHg%, *P* = 0.009; BVP: from 414.0 ± 241.5 to 291.8 ± 317.4 mmHg%, *P* = 0.005) segments in both groups. Myocardial Work differences between the lateral and septal segments was significantly reduced in both groups (LBBAP: from 1172.2 ± 563.5 to 633.1 ± 596.6 mmHg%, *P* = 0.001; BVP: from 877.4 ± 687.4 to −365.2 ± 644.9 mmHg%, *P* < 0.001) (Table 4). Compared with BVP, lateral segment MWE showed more improvement in the LBBAP group (3.5 ± 15.7 vs. −6.4 ± 22.9 mmHg% *P* = 0.006). Although it did not reach statistical significance, the MWE improvement in posterior segment was greater than that in the BVP group (3.8 ± 18.0 vs. −0.4 ± 17.5 mmHg%, *P* = 0.068) (Table 5).

## Discussion

This multicenter study evaluated the efficacy of LBBAP in advanced HF patients, focused mainly on mechanical synchronization and MW, and compared LBBAP with traditional BVP from an echocardiographic view. The major findings in our study cohort are as follows: (1) during the short-term observation, LBBAP was efficient in improving cardiac function, mechanical efficiency, and mechanical synchronization. (2) Our preliminary comparison between LBBAP and BVP showed that LBBAP resulted in greater improvement of mechanical synchronization and MW. To our knowledge, this is the first report which demonstrated the effects on mechanical synchronization and MW in patients with LBBAP.

### The Dilemma of CRT

Biventricular pacing is a well-established therapy for HF patients, but the non-response rate remains high. Although great efforts have been made to improve the response rate, the effect is far from satisfactory. This is partly due to limited mechanical dyssynchrony and MW (16). Permanent LBBAP was first reported by Huang et al. as a rescue pacing strategy after failure of CS-LV lead implantation, and significant improvement of LVEF and the clinical outcome was detected in their study during the 1-year follow-up (5). Recently, Zhang et al. reported that LBBAP induced great improvement of LVEF and LVESD in 11 consecutive HF patients during a short-term follow-up (17). An observational study conducted at Fuwai Hospital further demonstrated greater improvement in LVEF and electrical synchronization in the LBBAP group than in the BVP group (18). However, the study cohort was small, and little is known about the effect on mechanical synchronization and mechanical efficiency. It is not sufficient to draw the conclusion that LBBAP is superior to BVP in HF patients. Mechanical synchronization, which is quite important for cardiac pumping function, is not the same as electrical synchronization (19). Mechanical asynchrony and decreased mechanical efficiency will eventually result in HF and arrhythmia. Therefore, further study is needed to investigate its effect on mechanical synchronization and efficiency.

### The Efficacy of LBBAP in HF Patients

The duration of QRS has been accepted as a surrogate for predicting electrical synchronization (20). Salden et al. reported hemodynamic improvement and electrical resynchronization of LBBAP during short-term observation (21). A great reduction in QRSd was also detected in 61 LBBAP cases during the 1-year follow-up, in which the QRSd was improved from 169 ± 16 ms at baseline to 118 ± 12 ms (22). As shown in our study, the QRSd was significantly shortened in the LBBAP group compared with the baseline (from 177.1 ± 16.7 to 113.0 ± 18.4 ms), which reconfirmed that LBBAP can induce electrical synchrony. Furthermore, we evaluated mechanical synchrony using 2D-STI. The PSD was effectively shortened along with a shorter segment maximum time difference to peak 2-D strain in the LBBAP group during the observation, indicating its efficacy in improving interventricular mechanical synchronization in HF patients. Since LBBAP mainly activates the left bundle branch and results in right bundle branch conduction delay, there might be mechanical dyssynchrony between the ventricles. However, in our study, IVMD, which reflects intraventricular mechanical synchronization, was also effectively improved in the LBBAP group. This may be due to LBBAP being synchronized with intrinsic right bundle branch conduction and leading to further QRS narrowing and better intraventricular mechanical synchronization.

Mechanical efficiency was also evaluated in our study. The pressure-strain loop, which has emerged as a novel non-invasive method developed from STI, is efficient in quantitatively assessing mechanical synchrony and mechanical efficiency associated with LV pressure (11). As shown by Chan et al. (15), GWE was significantly reduced in HF patients, while GWE was high in normal hearts (23). Reduced GWE and excessive GWW may add an additional burden and contribute to myocardial remodeling. Galli et al. revealed that CRT responders have a significant improvement in LV synchrony along with a great increase in GWE and a reduction in GWW (24). Russell et al. validated its efficiency in evaluating the mechanical impact of dyssynchrony (12). In our study, LBBAP induced significant improvement in GWE and reduction in GWW. The increase in GWE and reduction in GWW might qualify for the restored ventricular systolic synchrony delivered by LBBAP. According to the European Association of Cardiovascular Imaging Normal Reference Ranges for Echocardiography (EACVI NORRE) study, the highest value for GWW among healthy subjects was 238 mmHg% in men and 239 mmHg% in women (25). In our study, the GWW in the LBBAP group was restored to 283.0 ± 129.6 mmHg%, which was close to normal.

In HF patients with CLBBB, abnormal electrical conduction leads to dyssynchronous ventricular contraction. Early contraction of the septum and systolic lengthening of the LV lateral wall may cause energy waste, which leads to inefficient mechanical wall motion (26). In previous research, significantly impaired septal wall MW was observed in HF patients with CLBBB, which may seriously impact LV functioning. Lateral wall MW was increased at the first stage of HF, and caused discordant LV contraction (27). Thus, restoring septum work and reducing septal-lateral work differences played an important role in patients' responses to CRT (28, 29). Prior to LBBAP, patients had inefficient septal function with markedly low MWE (35.3 ± 17.8%) and high WW (607.1 ± 276.5 mmHg%) compared with global LV (GWE:64.6 ± 7.8%;GWW: 410.3 ± 166.6 mmHg%).Regional analysis of MW revealed that LBBAP leads to significant MWE increases (from 35.3 ± 17.8 to 69.6 ± 19.0%, *P* = 0.001) and WW reductions (from 607.1 ± 276.5 to 330.8 ± 254.3 mmHg%, < 0.001) in the septum, indicating major improvement in LV function. Reducing septal-lateral work differences in the LV is an important determinant of reverse remodeling after CRT implantation (28, 30, 31). This study showed that LBBAP could reduce the regional differences in myocardial performance between the septal and lateral segments, which leads to more homogeneous regional MW distribution. From the perspective of MW, LBBAP induced great improvement in wall motion synchronization, which may result in better prognoses during long-term follow-up.

### Comparison Between LBBAP and BVP

A preliminary comparison between LBBAP and BVP revealed that LBBAP induced better improvement in specific echocardiographic parameters reflecting mechanical synchronization and MW and resulted in a relatively high echocardiographic response rate. Although the baseline MW in the groups was comparable and we used a linear mixed-effects model to reduce bias, this non-randomized study did not provide sufficient data to draw the conclusion that LBBAP is superior to BVP. However, analysis of segmental MW led to a better view of left ventricular motion patterns and mechanical synchronization. Biventricular pacing achieves ventricular mechanical synchronization by sequential RV and CS-LV lead pacing. This is not physiological, and the CS-LV lead is hard to implant in the region with the most delayed activation due to anatomical variability in coronary veins in clinical practice. In our study, BVP showed less efficacy in improving MWE and reducing WW in posterior and anterior segments, indicating its limited efficacy in achieving optimal synchronization of wall motion. This might be the reason why the non-response rate of BVP in our study was high. In the LBBAP group, the MWE tended to improve in every LV segment except for the lateral segment. Compared with BVP, LBBAP lead to better improvement of MWE in specific segments. Overall improvement of segmental MWE result in better GWE and better mechanical coordination. Moreover, LBBAP could activate the left bundle branch area. Thus, electrical stimulation passes down through the intrinsic conduction system instead of the intercellular electrical conduction, which may better maintain left ventricular systolic synchronization in HF patients.

## Conclusions

Left bundle branch area pacing was effective in improving cardiac function, mechanical synchrony and efficiency and may be a promising alternative CRT.

## Study Limitations

This is an observational study involving a limited number of participants. The follow-up period was short, and the long-term effects of LBBAP on cardiac function and mechanical synchronization need to be validated by more patients with longer follow-up periods. Although the clinical characteristics were comparable at baseline and we used a linear mixed-effects model with consideration of clinically relevant parameters to reduce the bias, this non-randomized study did not provide sufficient data to draw the conclusion that LBBAP is superior to BVP.

## Data Availability Statement

The raw data supporting the conclusions of this article will be made available by the authors, without undue reservation.

## Ethics Statement

The studies involving human participants were reviewed and approved by Zhongshan Hospital, Fudan University. The patients/participants provided their written informed consent to participate in this study.

## Author Contributions

WL and CH collected data, analyzed and interpreted the results, and drafted the manuscript. YW, YC, YZ, YL, and SZ collected and analyzed the data. XS and HC contributed to the design, planning, conduct of the study, and critically revised the manuscript. All authors contributed to the article and approved the submitted version.

## Conflict of Interest

The authors declare that the research was conducted in the absence of any commercial or financial relationships that could be construed as a potential conflict of interest.

## Publisher's Note

All claims expressed in this article are solely those of the authors and do not necessarily represent those of their affiliated organizations, or those of the publisher, the editors and the reviewers. Any product that may be evaluated in this article, or claim that may be made by its manufacturer, is not guaranteed or endorsed by the publisher.
