# Supplementary material for: Mechanical Synchrony and Myocardial Work in Heart Failure Patients With Left Bundle Branch Area Pacing and Comparison With Biventricular Pacing
Source: Front Cardiovasc Med. 2021 Aug 20;8:727611. doi: 10.3389/fcvm.2021.727611 (PMC8417592; doi:10.3389/fcvm.2021.727611)
Supplement: Supplementary file 1 [file Table_1.DOCX]

**Table S1. Changes in the BVP group at baseline and follow-up**

| **Variables** | **Baseline** | **Follow -up** | **Change** | **P value** |
| --- | --- | --- | --- | --- |
| **Cardiac function** |  |  |  |  |
| NYHA functional class | 2.8±0.6 | 2.2±0.8 | -0.9±0.8 | 0.001* |
| GLS(%) | -6.2±2.7 | -8.5±3.5 | -2.3±2.6 | <0.001* |
| LVEF(%) | 29.5±4.9 | 43.1±11.0 | 13.7±11.5 | <0.001* |
| **Synchronization** |  |  |  |  |
| QRSd（ms） | 168.8±16.8 | 136.3±20.1 | -32.5±22.3 | <0.001* |
| IVMD (ms) | 60.3±25.4 | 41.7±24.2 | -18.6±27.9 | <0.001* |
| PSD(ms) | 138.4±43.1 | 111.5±50.5 | -26.9±63.9 | 0.018* |

NYHA=New York heart association; GLS= global longitudinal strain ;LVEF=left ventricular ejection fraction ；

IVMD= Interventricular mechanical delay; PSD= Peak strain dispersion; * p＜0.05
